# Effects of flaxseed germination on oil composition, tissue n-3 fatty acid profiles, and brain lipidomics in mice

**DOI:** 10.1016/j.fochx.2026.104654

**Published:** 2026-10-18

**Authors:** Zeqing Huang, Shuai Xu, Yonglin Li, Keqiang Huang, Chongyang Liu, Yuan Gao, Qi Li, Xiuzhu Yu

**Affiliations:** a Shaanxi Union Research Center of University and Enterprise for Functional Oil Engineering Technology, College of Food Science and Engineering, Northwest A&F University, 22 Xinong Road, Yangling 712100, Shaanxi, PR China; b Institute of Agricultural Products Processing Engineering Research, Academy of Agricultural Planning and Engineering, Ministry of Agriculture and Rural Affairs, Maizidian Street No.41, Chaoyang District, 100125, Beijing, China; c Shanxi Liming Grain, Oil and Trade Co., Ltd, Hanzhong 723500, China; d Yangling Qinyang Agricultural Technology Co., Ltd., Yangling 712100, China

**Keywords:** Flaxseed oil, Germination, α-Linolenic acid, EPA, DHA, Lignan

## Abstract

Germination can enrich bioactive components in flaxseed oil, but its effects on tissue lipid profiles remain unclear. This study examined changes in oil composition and tissue lipid responses to germinated flaxseed oil (GFO). Mice received soybean oil, ungerminated flaxseed oil (UGFO), or GFO for 13 weeks. Adipose tissue and red blood cell fatty acid profiles and brain lipidomics were assessed. GFO contained 1.3 times the sterol content and 3.4 times the lignan content of UGFO, but its alpha-linolenic acid (C18:3n-3, ALA) proportion was lower (53.35% versus 57.17%). Most measured tissue n-3 fatty acid outcomes showed no significant differences between GFO and UGFO. Brain lipidomics revealed differences in several EPA- and DHA-containing species relative to soybean oil controls. Our findings characterize the tissue lipid responses to UGFO and GFO, and provides a basis for optimizing germination through a balance between ALA retention and minor component enrichment.

## Highlights

• Germination enriched lignans and sterols but lowered the oil ALA proportion.
• A 13-week flaxseed oil dietary intervention with flaxseed oil was conducted in mice.
• Most tissue n-3 fatty acid measures showed no significant GFO-UGFO differences.

## 1 Introduction

Flaxseed (*Linum usitatissimum* L.) is an important oilseed crop cultivated worldwide. It is also known as linseed. Flaxseed oil is a widely consumed vegetable oil valued for its distinctive fatty acid profile. Flaxseed oil constitutes one of the richest dietary sources of n-3 polyunsaturated fatty acids (PUFAs) (Wu et al., 2019). The PUFAs may account for up to 70% of the total fatty acid content in flaxseed oil (Yang et al., 2021). Among these PUFAs, α-linolenic acid (C18:3n-3, ALA) is of particular interest, as it is an essential fatty acid that can not be synthesized by the human body. Moreover, ALA serves as a dietary precursor to n-3 PUFAs and can be converted to eicosapentaenoic acid (20:5n-3, EPA) and docosahexaenoic acid (22:6n-3, DHA) in vivo. The EPA and DHA are critical n-3 PUFAs that contribute to neural development during infancy and adolescence, cardiovascular protection, and the mitigation of Alzheimer's disease risk (Punia et al., 2019). However, as EPA and DHA are predominantly found in seafood, individuals with limited access to or low consumption of seafood are at increased risk of deficiency (Micha et al., 2014). Therefore, flaxseed oil rich in ALA represents a valuable alternative source of n-3 PUFAs for individuals unable to obtain sufficient EPA and DHA from marine sources.

The essential fatty acid ALA serves as a precursor for long-chain n-3 PUFAs through sequential desaturation and elongation reactions. ALA is initially desaturated by Δ6-desaturase (FADS2), elongated, and subsequently desaturated by Δ5-desaturase (FADS1) to form EPA. Further reactions involving ELOVL2/ELOVL5 elongases and FADS2 generate C24:6n-3, which undergoes one cycle of peroxisomal β-oxidation to yield DHA (Kim et al., 2014; O'Neill et al., 2021). This biosynthetic capacity varies among tissues. In mice, the liver exhibits the highest capacity among the tissues examined, whereas the brain, testes, and kidneys show more limited activity (Valenzuela et al., 2024). Conversion also depends on substrate availability and the nutritional and metabolic regulation of the participating enzymes. Insulin and PUFA availability can regulate desaturase and elongase expression, while oxidative stress may alter their expression and activity and promote PUFA loss through lipid peroxidation (Videla et al., 2022). Therefore, dietary ALA availability alone does not determine tissue EPA and DHA status. Studies also demonstrated that supplementation of flaxseed or flaxseed oil in human and mice significantly elevates the ALA and EPA content in plasma and serum (Edel et al., 2016; Harper et al., 2006; James et al., 2003). However, the majority of these investigations quantified fatty acids exclusively in plasma or serum and failed to detect a concomitant rise in DHA following ALA, flaxseed, or flaxseed-oil consumption (Fan et al., 2020). Because DHA is predominantly deposited in brain tissue, the impact of flaxseed oil intake on cerebral DHA status remains unclear.

Germination is a crucial phase in flaxseed life cycle, with water imbibition by dry flaxseed and elongation of the embryonic axis. Concomitantly, metabolic pathways in flaxseed are extensively activated, including mobilization of stored sugars and lipids, to supply energy for protein synthesis and other anabolic processes required for subsequent growth. Among these metabolites, flaxseed lignan represents a major class of phenolic compounds exhibiting diverse bioactivities, including antioxidant, anti-inflammatory, and cholesterol-lowering effects, as well as amelioration of diabetes, depression, bone loss, and menopausal symptoms, and enhancement of athletic performance (Sangiorgio et al., 2023; Westcott & Muir, 2003). These lignans are characterized by diphenolic ring structures and occur in both free and glycosidic forms (Hu et al., 2024). Most importantly, accumulating evidence indicated that flaxseed lignans facilitated the conversion of ALA to EPA and DHA through up-regulating elongase and desaturase, while inhibiting β-oxidation related enzymes (Drygalski et al., 2017; Mellery et al., 2017; Wang et al., 2025). Our previous work has demonstrated that germination induced massive synthesis of phenolic compounds and concomitantly enhanced the antioxidant capacity of both flaxseed and flaxseed oil. However, the variation of specific flaxseed lignan in ungerminated flaxseed oil (UGFO) and germinated flaxseed oil (GFO) remain to be elucidated. Additionally, previous studies addressing flaxseed lignan facilitated the conversion of ALA were conducted either in vitro using cell models (Drygalski et al., 2017; Mellery et al., 2017) in vivo over a relatively short 35 d animal trial (Wang et al., 2025). The effect of GFO with elevated lignan content on the ALA, EPA and DHA in tissue remains unclear.

This study aimed to determine how germination alters flaxseed oil composition and whether consumption of germinated flaxseed oil affects tissue n-3 fatty acid profiles and brain lipid composition compared with ungerminated flaxseed oil. UGFO and GFO were characterized for their fatty acid, sterol, and lignan compositions and evaluated in a 13-week dietary intervention in male C57BL/6 J mice, with soybean oil serving as the control fat source. Fatty acid profiles in adipose tissue and red blood cells, together with brain lipidomics, were assessed to examine the tissue responses to these dietary oils. The findings were intended to inform the optimization of germination conditions by considering both oil composition and its nutritional implications.

## 2 Materials and methods

### 2.1 Materials

Flaxseed (*Linum usitatissimum* L.) specimens were procured from Inner Mongolia Yiyuan Biotechnology Co., Ltd. (Inner Mongolia, China). Sodium hydroxide, potassium hydroxide, and hydrochloric acid were analytical grade, purchased from Tianjin Chemical Co., Ltd. (Tianjin, China). Ethanol, methanol, and methyl tert-butyl ether (MTBE) were HPCL grade, as well as *N*,*O*-Bis(trimethylsilyl)trifluoroacetamide with 1% trimethylchlorosilane (BSTFA+1% TMCS), BF_3_, and 1 × TE buffer (pH = 7.4) were purchased from Macklin Biochemical Technology Co., Ltd. (Shanghai, China). Standard of 5α-Cholestan-3β-ol was acquired from Aladdin Biochemical Technology Co., Ltd., (Shanghai, China). Standards of pinoresinol diglucoside (PDG), secoisolariciresinol diglucoside (SDG), secoisolariciresinol (SECO), and pinoresinol (PINO) were purchased from ChemFaces Co., Ltd. (Wuhan, China). the purity of all standards was higher than 98% and guaranteed by suppliers.

### 2.2 Flaxseed germination

The germination procedure was performed according to our previous study (Dong et al., 2023) with minor modifications. A total of 10 kg of flaxseeds was randomly divided into three independent batches. Each batch included an ungerminated control and a germination treatment, providing three independent replicates for each treatment. Flaxseeds were manually sorted to remove impurities and damaged seeds, then thoroughly rinsed with deionized water. Seeds were subjected to 4 successive washing/steeping cycles (30 °C, 15 min each) with fresh deionized water to rapidly break dormancy. After complete drainage of the soaking medium, seeds were transferred to a germination chamber maintained in darkness. Two layers of sterile filter paper were placed on the chamber floor; conditions were set to 25 °C and 80% relative humidity. Based on our previous study (Zhang et al., 2023), a 4 d germination period was chosen to optimize the tradeoff between oil yield and phenolic content. Germinated seeds were harvested and dried at 40 °C for 8 h to terminate metabolic activity.

### 2.3 Preparation of ungerminated and germinated flaxseed oil

According to our previous study (Zhang et al., 2023), the ungerminated and germinated seeds from each batch were roasted at 160 °C for 15 min and screw-pressed separately after cooling, yielding three independently prepared UGFO samples and three independently prepared GFO samples. Crude oils were centrifuged at 8000 ×*g* to remove other solid particles. The acid value and peroxide value of ungerminated flaxseed oil (UGFO) and germinated flaxseed oil (GFO) were quantified following the AOCS official method Cd 3d-63 (AOCS, 2009a) and Cd 8b-90 (AOCS, 2009b), respectively. Oils were stored at 4 °C.

### 2.4 Determination of fatty acid profile of ungerminated flaxseed oil (UGFO) and germinated flaxseed oil (GFO)

Fatty acid profile determination followed protocol of Zhang et al. (2023) with modification, briefly, 0.1 g oil was methyl esterified by methanol and BF_3_. After extraction and dehydration, samples were analyzed through gas chromatography-flame ionization detector (GC-FID, Nexis GC-2030, Shimadzu, Japan). The GC-FID equipped with SH-Rt-2560 capillary column (100 m × 0.25 mm I.D., 0.2 μm film thickness). GC-FID parameter settings followed the Chinese national standard GB 5009.168–2016. Fatty acids were qualified by the retention time of 37 fatty acid methyl esters standard (TMRM Standard Substance Co., Ltd., Jiangsu, China). Fatty acid percentage was quantified through peak area normalization method.

### 2.5 Determination of sterols in UGFO and GFO

Sterols in UGFO and GFO was determined through the method described by Li et al. (Li et al., 2023). Briefly, 20 μL of 0.5 mg/mL 5α-cholestan-3β-ol solution (internal standard) was added to 0.015 g oil, and was saponified using 2 M KOH/95% EtOH. Then sterols were extracted by *n*-hexane. After condensation and derivatization through BSTFA+1% TMCS, sterols were analyzed by GC–MS. A 6890 N/5973 inert GC–MS system (Agilent, Santa Clara, USA) equipped with an DB-5MS Ultra Inert capillary column (30 m × 0.25 mm I.D., 0.25- μ m film thickness, Agilent, Santa Clara, USA) was used for sample determination. The derivatized sample (1 μ L) was injected into the oven with the split mode at a split ratio of 20:1. The temperature of the injector was set at 320 °C. The GC oven temperature was programmed as follows: 180 °C for 1 min, 4 °C min^−1^ ramped to 300 °C, and held at 300 °C for 15 min. The total GC run time of analysis was 46 min per sample. Ultrapure-grade helium was employed as a carrier gas at a flow rate of 0.7 mL/min. Selected ion mode was applied to quantify sterol content. The quantifier ion and qualifier ions of each compound are shown in Table 1. The analytes were ionized by electron impact, with 70 eV of ion energy. The solvent delay time was 10 min. The temperatures of transfer line and ion source were 280 °C and 250 °C, respectively. Content of sterols was calculated from calibration curves established using sterol standard substances.

**Table 1:** Quantifier and qualifier ions of internal standard and sterols.

Compound	Quantifier ion	Qualifier ions
5α-cholestan-3β-ol	215.2	355.4, 305.3
Campesterol	343.4	382.4, 367.4
Stigmasterol	394.4	255.3, 484.5
β-Sitosterol	357.5	396.5, 381.5
Δ5-Avenasterol	296.3	469.5, 257.3
Cycloartenol	365.4	339.4, 408.5

### 2.6 Determination of lignans in UGFO and GFO

The standards of pinoresinol diglucoside (PDG), secoisolariciresinol diglucoside (SDG), secoisolariciresinol (SECO), and pinoresinol (PINO) were made to 100 mg/L methanol single standard solutions and 10 mg/L methanol mixed standard solution. Single standard solutions were used to determine the retention times of each lignan, and the mixed standard solution was further diluted to 0.5, 1, 2, 4, 8, 16, 32, and 48 mg/L to establish the calibration curves. Solutions of 0.5, 3, 18, and 40 mg/L was prepared as quality control samples to determine spike recoveries and RSD.

For sample determination, 0.3 g of oil was weighed and 5 mL 70% ethanol solution containing 1 M NaOH was added. After vortex, the mixture was subjected to 700 W ultrasonic treatment at 65 °C for 30 min. Then the mixture was centrifugated with 5000 ×g for 10 min. The pH of upper layer was adjusted to 5.5–6.0 using hydrochloric acid followed by adjusting the volume of solution to 25 mL.

A LC-40D ultra performance liquid chromatography-photo-diode array (UPLC-PDA, Shimadzu, Japan) was used to analyze lignans. The UPLC-PDA equipped with CORTECS Premier C18 Column (2.7 μm particle size, 2.1 mm × 150 mm) and CORTECS C18 VanGuard Cartridge (2.7 μm particle size, 2.1 mm × 5 mm, Waters, USA). The injection volume was 2 μL, mobile phase A was water of HPLC grade, mobile phase B was methanol. The elution process was 80% mobile phase A at 0 min, then decreased to 45% mobile phase A at 5 min, followed by further decreased to 20% mobile phase A at 10 min. To 13 min, the percentage of mobile phase A was decreased to 5%. In 13–18 min, the mobile phase A increased to 80%. The column oven temperature was 35 °C, and flow rate of mobile phase was 0.39 mL/min. Wavelengths used to quantify the lignans were: 227 nm for PDG and SECO, 230 nm for SDG, and 280 nm for PINO. Content of lignans was determined though external standard method and calculated from calibration curves.

### 2.7 Preparation of animal chow

Three batches of UGFO and GFO were combined and incorporated into isocaloric experimental chows for mice. Three isocaloric diets based on a modified AIN-76 A formulation were prepared using soybean oil, UGFO, or GFO as the added fat source for groups C, N, and G, respectively. According to the supplier's formulations, each diet contained 108.4 g of the corresponding oil per 927.05 g of diet, equivalent to approximately 11.7% (*w*/w). Protein, carbohydrate, and fat provided 20.8%, 54.2%, and 25.0% of total energy, respectively. The diet formulations are presented in Table S1. The fatty acid composition of the soybean oil was determined using the method described in Section 2.4, and the fatty acid compositions of all three dietary oils are summarized in Table S2. ALA represented 6.29%, 57.17%, and 53.35% of total fatty acids in soybean oil, UGFO, and GFO, respectively. All diets were stored at −20 °C.

### 2.8 Animal experiment assay

#### 2.8.1 Design of animal experiment

A 13-week dietary intervention experiment was carried out in eight-week-old male C57BL/6 J mice. The mice were randomly assigned to 3 groups (*n* = 12): control group (C) receiving modified AIN-76 A rodent diet (soybean), the ungerminated flaxseed oil group (N) receiving modified AIN-76 A rodent diet (UGFO), and the germinated flaxseed oil group (G) receiving modified AIN-76 A rodent diet (GFO), shows in Fig. S1.

#### 2.8.2 Animals

The experiment was conducted with six-week-old male C57BL/6 J mice, which were procured from Chengdu DOSSY experimental animals Co., Ltd. (Chengdu, China). The supplier guaranteed the genetic purity of the mouse strain. Mice were housed under controlled conditions: room temperature (25 ± 1 °C), relative humidity (60–75%), and a 12-h:12-h light–dark cycle (lights on at 06:00 h). All procedures in this study complied with the guidelines outlined in the ‘Guide for the Care and Use of Laboratory Animals: Eighth Edition’ (ISBN-10: 0–309–15,396-4) and American Veterinary Medical Association guidelines for the euthanasia of animals: 2020 edition (AVMA, 2020). The animal experiment was approved by the Faculty Animal Policy and Welfare Committee of Northwest A&F University (Permission ID: IACUC 2025–0505).

#### 2.8.3 Animal experiment assay

After acclimation for 2 weeks, mice were randomly assigned to groups and subjected to a 13-week dietary intervention. Body weight was measured weekly, and cheek blood samples were collected every 2 weeks. At week 13, mice were euthanized by carbon dioxide asphyxiation, followed by aortic exsanguination. After collecting blood, the mice epididymal fats, mesenteric fats, inguinal white adipose tissue, and brain were collected. Blood samples was centrifuged with 2000 ×*g* for 15 min. The plasma (heparin sodium as anticoagulant), RBC, and collected tissues were stored at −80 °C for subsequent analysis.

#### 2.8.4 Determination of plasma total triglycerides (TG), total cholesterol (TC), low density lipoprotein cholesterol (LDL), and high density lipoprotein cholesterol (HDL)

Determination of plasma total triglycerides (TG), total cholesterol (TC), low density lipoprotein cholesterol (LDL-C), and high density lipoprotein cholesterol (HDL—C) was processed using colorimetric assay kit (Elabscience Biotechnology Co., Ltd., Wuhan, China). Plasma of 4 mice in a group was pooled. Pooled plasma was subjected to determine TG, TC, LDL-C, and HDL-C (*n* = 3 per group) following the instructions of kit.

#### 2.8.5 Determination of fatty acid profile of tissues fat and red blood cells (RBC) lipid

For each mouse, the epididymal, mesenteric, and inguinal adipose tissues collected as described in Section 2.8.3 were combined. Adipose tissues from different mice were processed separately, with each mouse considered an independent biological replicate (*n* = 12 per group). For every 50 mg of tissue, 200 μL of deionized water was added. The mixture was homogenized through Tissuelyser-96 L tissue homogenizer (Shanghai Jingxin Industrial Development Co., Ltd., Shanghai, China). Then, 400 μL MTBE and 80 μL methanol were added and vortexed. After 700 W ultrasonic treatment for 10 min, the mixture was centrifugated with 8000 ×g for 10 min. The upper organic layer was dried by nitrogen flow to obtain tissue fat. For RBC lipid extraction, the collected RBC in 2.8.3 was resuspended by 1 mL TE buffer and incubated on ice for 10 min. The mixture was centrifugated with 15,000 ×g for 8 min and the supernatant was discarded. This washing step, addition of TE buffer followed by centrifugation, was repeated until the pellet appeared milky white. To every 100 μL lower layer, 340 μL extractant (MTBE: methanol: water = 10:2:5, v:v:v) was added and vortexed. Then the mixture was then subjected to 700 W ultrasonication for 10 min, followed by centrifugating with 8000 ×g for 10 min. The upper layer was dried by nitrogen flow to obtain RBC lipids. The fatty acid profile of adipose tissue lipids and RBC lipids were analyzed according to the procedure described in section 2.4.

#### 2.8.6 Brain lipidomics

Six brains were randomly selected in each group to conduct **lipidomics** analysis. Lipids were extracted from approximately 30 mg of frozen tissues using a modified version of the Bligh and Dyer's method as described previously (Lam et al., 2025; Miao et al., 2022). Briefly, tissues were homogenized in 900 μL of chloroform: methanol: water (3:6:1, v:v:v). The homogenate was then incubated at 1500 rpm for 30 min at 4 °C. At the end of the incubation, 350 μL of deionized water and 300 μL of chloroform were added to induce phase separation. The samples were then centrifuged and the lower organic phase containing lipids was extracted into a clean tube. Lipid extraction was repeated once by adding 500 μL of chloroform to the remaining aqueous phase, and the lipid extracts were pooled into a single tube and dried under nitrogen flow. Samples were stored at −80 °C until further analysis.

Lipidomic analyses were conducted at LipidALL Technologies using a Jasper HPLC coupled with Sciex TRIPLE QUAD 4500 MD (AB Sciex Co., Framingham, USA) as reported previously (Lam et al., 2021). Separation of individual lipid classes by normal phase (NP)-HPLC was carried out using a TUP-HB silica column (3 μm particle size, 150 × 2.1 mm, Tuplabs Co., Ltd., Tianjin, China) with the following conditions: mobile phase A (chloroform: methanol: ammonium hydroxide, 89.5:10:0.5) and mobile phase B (chloroform: methanol: ammonium hydroxide: water, 55:39:0.5:5.5). Multiple reaction monitoring transitions were set up for comparative analysis of various lipids. Individual lipid species were quantified by referencing to spiked internal standards. d9-PC32:0 (16:0/16:0), PE 34:0, dic8-PI, d31-PS, C17:0-PA, DMPG, CL-14:0, C14-BMP, C12-SL, C17-LPC, C17:1-LPI, C17:0-LPA, C17:1-LPS, C17-Cer, C12-SM, C8-GluCer, C8-LacCer, d3–16:0 carnitine, DAG (18:1/18:1)-d5 were obtained from Avanti Polar Lipids (Richmond, USA). GM3-d18:1/18:0-d3 was purchased from Matreya LLC (Ann Arbor, USA). Free fatty acids were quantitated using d31–16:0 (Sigma-Aldrich, Merck KGaA, Darmstadt, Germany). d6-18 CE:0 and TAG (16:0)3-d5 were obtained from CDN isotopes (Ontario, Canada).

### 2.9 Statistical analysis

The results are expressed as the mean ± SD. One-way ANOVA and multiple comparisons (Tukey test) were performed with Minitab 18.1 (Minitab, Inc., USA). *p* < 0.05 was considered to indicate statistical significance. Data plotting was performed via Origin Pro 2021 (Originlab Co., USA) and cloud platform of LipidALL Technologies Co., Ltd. (Changzhou, China).

## 3 Results and discussion

### 3.1 Fatty acid profile of UGFO and GFO

The fatty acid profile of UGFO and GFO is presented in Table 2. The UGFO containing rich unsaturated fatty acid, particularly ALA (57.17%), demonstrates the high nutritional value of flaxseed oil. Our findings regarding the fatty acid composition of UGFO were consistent with those reported by Danish and Nizami (2019). The ALA can be converted to other physiologically important n-3 polyunsaturated fatty acids in vivo, such as EPA, DPA, and DHA, etc. Thus, flaxseed oil with a high ALA content may help mitigate EPA deficiency in populations with limited access to fish. Germination has been shown to enhance the levels of bioactive compounds as well as antioxidant and antiproliferative activities (Li et al., 2019; Wang et al., 2016). However, after the 4 d of germination, the proportion of ALA significantly decreased from 57.17% to 53.35%, similarly, linoleic acid (C18:2, LA) declined from 16.14% to 15.52%. These findings are consistent with previous reports. In a prior study by our group, the ALA content in flaxseed decreased significantly after 5 d of germination (Dong et al., 2023). In another study, ALA content in 2 of 3 flaxseed cultivars decreased significantly after 4 or 5 d of germination (Zhang et al., 2023). This reduction is attributed to the high energy demand during germination, long chain fatty acids are β-oxidized to generate acetyl coenzyme A. (Kalish et al., 2013; Yaniv et al., 1998). Correspondingly, the decreases in ALA and linoleic acid were accompanied by increases in the relative proportions of palmitic acid, stearic acid, and oleic acid. These compositional changes raised the question of whether the lower ALA proportion in GFO would affect tissue n-3 fatty acid status following long-term consumption. Because germination may also alter minor oil components involved in fatty acid metabolism, sterols and lignans in UGFO and GFO were subsequently characterized, followed by a dietary intervention study in mice.

**Table 2:** Fatty acid profile of UGFO and GFO.

Fatty acid (%)^a^	UGFO	GFO
C16:0	5.43 ± 0.08	5.55 ± 0.02
C18:0	3.96 ± 0.04	4.85 ± 0.01*
C18:1n-9	17.30 ± 0.11	20.73 ± 0.03*
C18:2n-6	16.14 ± 0.03	15.52 ± 0.003*
C18:3n-3	57.17 ± 0.27	53.35 ± 0.06*

a : “*”after the fatty acid percentage of germinated flaxseed oil (GFO) means significant difference of same fatty acid among ungerminated flaxseed oil (UGFO) and GFO.

### 3.2 Sterol content of UGFO and GFO

Sterols are class of compounds characterized by a perhydrocyclopentanophenanthrene ring structure, which is structurally analogous to that of cholesterol. Thus, they have function to reduce the absorption of cholesterol. In addition, sterols show bioactivities of anti-inflammation (Feng et al., 2017) and anti-oxidation (Zhao et al., 2019). In terms of lipid absorption and conversion, sterols can activate enzymes for synthesizing fatty acid (Wollam & Antebi, 2011) and inhibit enzymes for catalyzing oxidation of fatty acid (Laraki et al., 1993). The content of main 5 sterols in flaxseed oil are depicted in Fig. 1a. After 4 d of germination, the total sterol concentration in flaxseed oil increased. The concentration of cycloartenol increased significantly from 25.65 to 33.07 mg/kg, consistent with findings from previous studies (Xie et al., 2025; Zhang et al., 2023). During germination, flaxseed undergoes rapid cellular development, necessitating the synthesis of new cell membranes (Du et al., 2022). The observed increase in sterol levels may be attributed to their essential role as structural components of cell membranes.

**Fig. 1:**
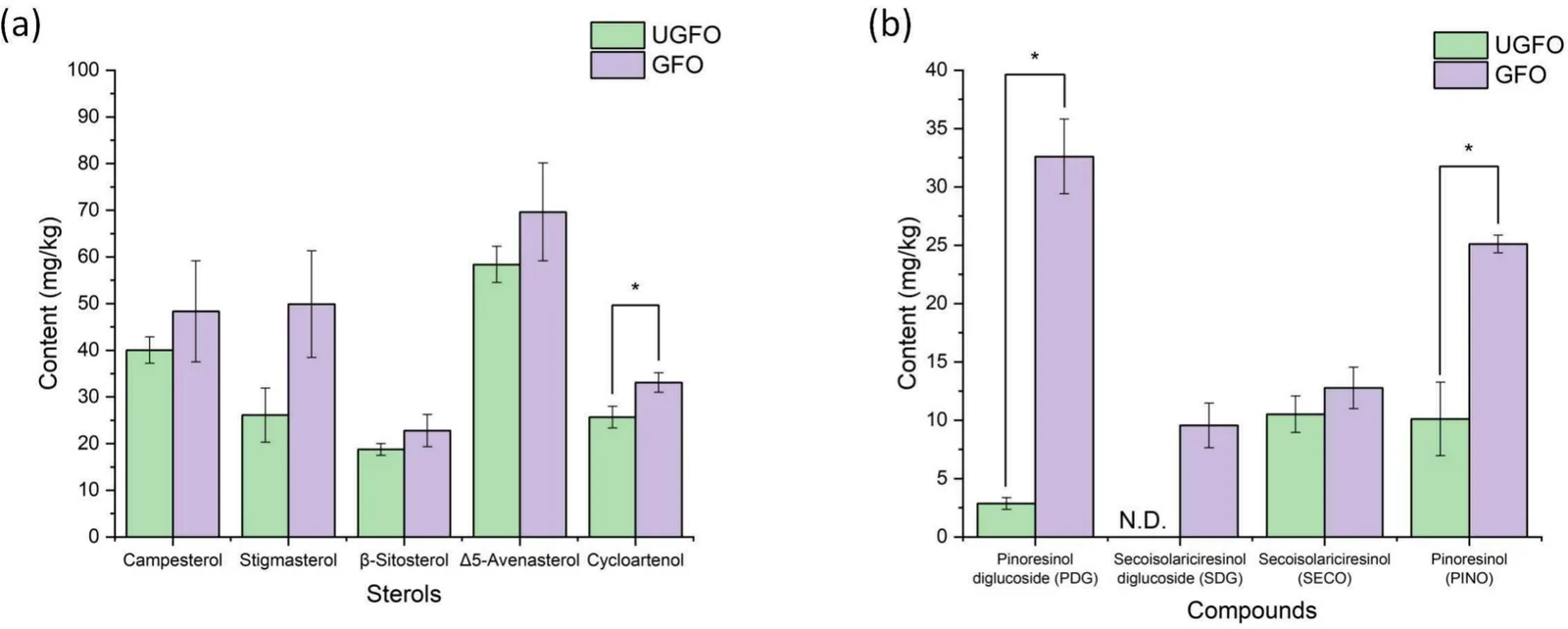
Contents of sterols (a) and lignans (b) in UGFO and GFO. Values are expressed as the mean ± SD of three independently prepared oil samples (*n* = 3). One-way ANOVA was performed between UGFO and GFO, and * indicates a significant difference (*p* < 0.05).

### 3.3 Lignans content of UGFO and GFO

Lignans are the most important bioactive phenolic compounds in flaxseed oil. They are diphenolic molecules composed of two phenylpropanoid units. Lignans can exist in both free and glycosidic forms (Hu et al., 2024). The presence of two phenolic hydroxyl groups confers lignans their potent antioxidant activity. Moreover, lignans exhibit a range of biological activities, including anticancer effects, modulation of hormonal balance, and amelioration of cardiovascular, renal, and metabolic disorders such as diabetes (Westcott et al., 2003), as well as estrogenic, anti-estrogenic, and antidepressant properties (Sangiorgio et al., 2023). The primary lignans in flaxseed oil include PDG, SDG, SECO, and PINO. Method was developed based on UPLC-PDA, which used to determine the content of lignans in oil. Quality control result of spike recovery was ranged from 85% to 115%, RSDs were lower than 15% (data not shown). Then the content of lignans in UGFO and GFO was determined, as shown in Fig. 1b.

The content of lignans in flaxseed oil increased dramatically after germination. In UGFO, PDG was present at 2.86 mg/kg, germination increased its concentration to 32.60 mg/kg. The free form of PDG, i.e. PINO, it increased from 10.11 mg/kg to 25.10 mg/kg. Regarding SECO, it exhibited no significant difference between UGFO and GFO. However, the glycosidic form of SECO, i.e. SDG, had not been detected in UGFO. In GFO, SDG was quantified at 9.56 mg/kg. These results are consistent with those of Zhang et al. (2023) who reported significant increases in total phenolic content and antioxidant capacity after 4 days of germination across all 3 flaxseed cultivars examined. The increase in lignan levels may be attributed to the mobilization of stored lipids, carbohydrates, and proteins during germination, which releases phosphoenolpyruvate (PEP) and free amino acids for de novo protein synthesis. The PEP activates the shikimate pathway to produce aromatic amino acids; in conjunction with pre-existing free aromatic amino acids, this enhances lignan biosynthesis (Dong et al., 2023). Overall, the increasing extent of glycosidic lignans was higher than that of free form. This may be due to the release of reducing sugars during germination, which facilitates the glycosylation of lignans (Dong et al., 2024). Many studies showed that lignans facilitated the absorption and conversion of ALA to EPA and DHA in vivo (Drygalski et al., 2017; Ide et al., 2015; Mellery et al., 2017; Tsuruoka et al., 2005). Therefore, we conducted the long-term dietary intervention on mice to evaluate the effect of GFO on the ALA, EPA, and DHA of tissue in the case of its reduced ALA content.

### 3.4 Body and adipose tissue weight, TG, TC, LDL-C, and HDL-C of mice

Three groups of mice, namely C, N, and G were fed a modified AIN-76 A rodent diet (soybean), modified AIN-76 A rodent diet (UGFO), and modified AIN-76 A rodent diet (GFO), respectively. After a 13-week of dietary intervention, the body and adipose tissue weight, TG, TC, LDL-C, and HDL-C were assessed, with results presented in Fig. 2.

**Fig. 2:**
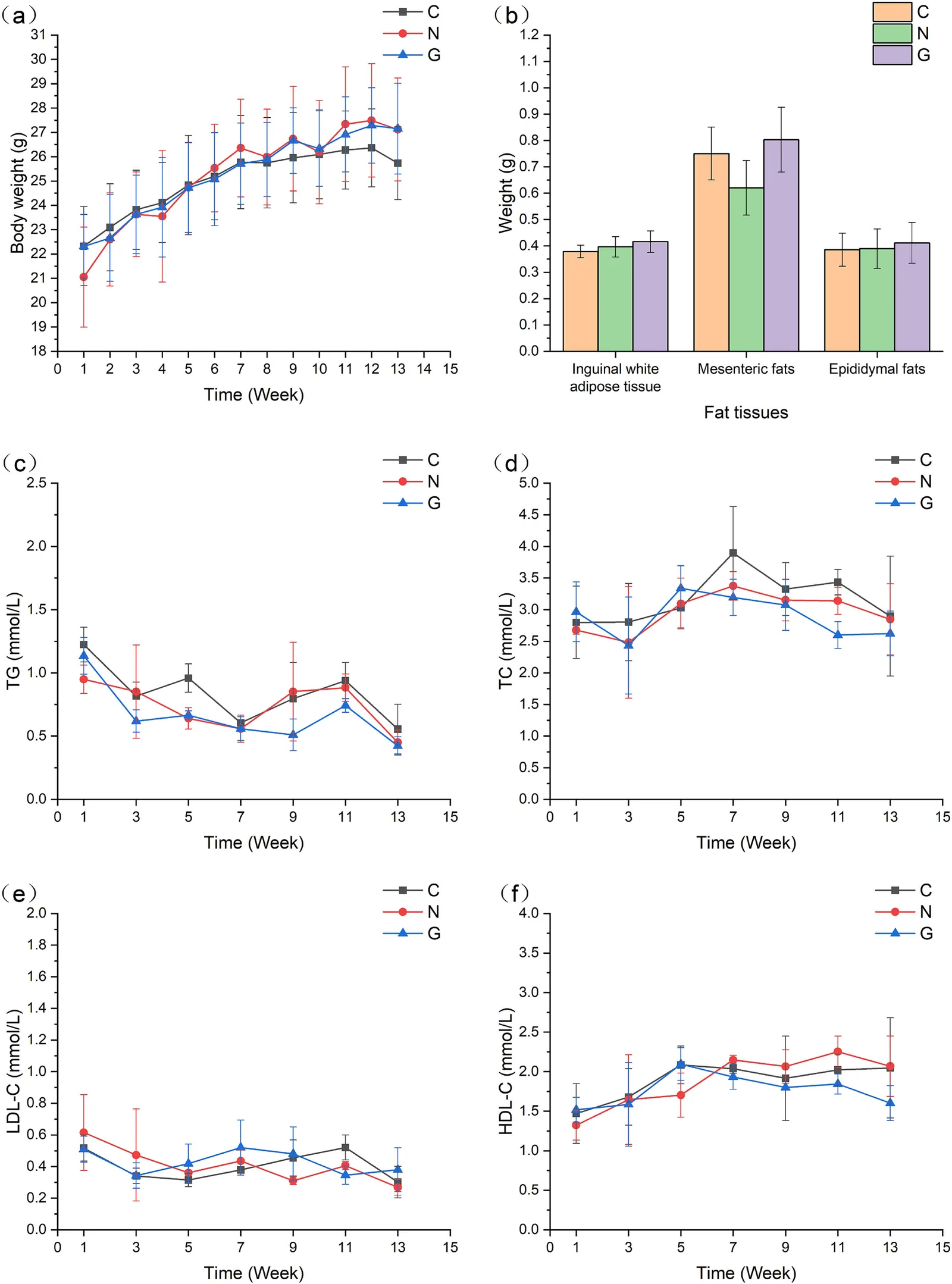
Body weight (*n* = 12) (a), adipose tissue weight (*n* = 12) (b), total triglycerides (TG, c), total cholesterol (TC, d), low density lipoprotein cholesterol (LDL-C, e), and high density lipoprotein cholesterol (HDL—C, f) of mice in 3 groups (plasma of 4 mice was pooled, *n* = 3). All values are represented as the mean ± SD. One way ANOVA was performed among 3 groups withTukey's multiple comparison.

During the dietary intervention, body weight increased in all 3 groups, as shown in Fig. 2a. during the first 7 weeks, mice exhibited rapid body weight gain. Thereafter, the rate of body weight gain gradually declined. This growth pattern is consistent with that previously reported for C57BL/6 J mice (Yang et al., 2014). No significant differences were found among 3 groups, indicating the 3 groups of diets had not induced obesity or malnutrition. This conclusion was further supported by adipose tissue mass, which showed no significant differences among the 3 groups (Fig. 2b) and aligned with findings from prior studies (Atal et al., 1994; Sakane et al., 2021; Wamil et al., 2011). Collectively, body and adipose tissue weights indicated normal metabolic status in all 3 groups, which was further corroborated by plasma biochemical parameters. Values of TG, TC, LDL-C, and HDL-C are illustrated in Fig. 2c-2f. All biochemistry values fell within normal ranges (Fan et al., 2020; Otto et al., 2016), and no significant intergroup differences were observed, confirming normal metabolic function across all groups. The maintenance of normal metabolic status ensured the reliability of subsequent fatty acid profiling and lipidomic analysis.

### 3.5 Fatty acid profile of adipose tissue and RBC

The fatty acid profiles of adipose tissues combined within each mouse are presented in Table 3. Compared with group C, groups N and G showed significantly higher ALA proportions and lower linoleic acid proportions. Despite the lower ALA proportion in GFO, adipose tissue ALA proportions did not differ significantly between groups N and G. RBC fatty acid profiles were subsequently examined to assess the response in another lipid compartment (Fig. 3).

**Table 3:** Fatty acid profile of adipose tissue of 3 groups^a^*.*

Fatty acid (%)^b^	Group C	Group N	Group G
C16:0	21.00 ± 2.55	20.97 ± 0.77	20.81 ± 2.42
C18:0	1.88 ± 0.81	1.71 ± 0.18	1.86 ± 0.95
C18:1n-9	32.18 ± 1.62	31.77 ± 0.49	33.18 ± 2.88
C18:2n-6	34.46 ± 6.53a	14.95 ± 0.37b	16.47 ± 6.23b
C18:3n-3	4.30 ± 6.89b	22.58 ± 0.78a	20.53 ± 6.80a

a : Values are expressed as the mean ± SD (*n* = 12 mice per group). For each mouse, epididymal, mesenteric, and inguinal adipose tissues were combined and analyzed.

b : Same letter after data of a fatty acid means no significant difference (*p* > 0.05) between groups.

**Fig. 3:**
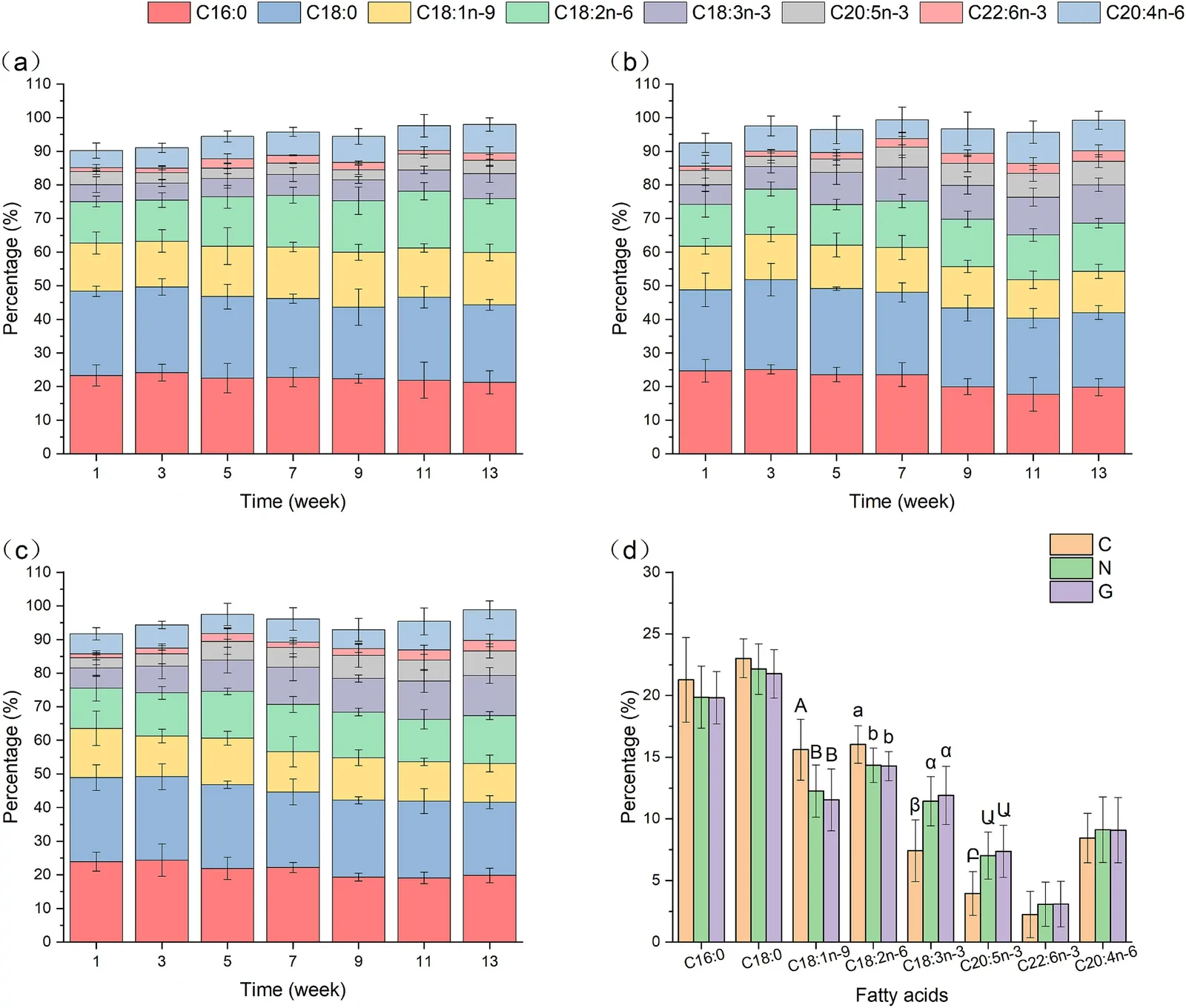
Fatty acid profile variation of RBC lipid in group C (a), group N (b), and group G (c), and percentage of fatty acids between group C, group N, and group G at 13th week (d). In (d), One way ANOVA was performed among 3 groups withTukey's multiple comparison. Fatty acid abbreviations: C16:0, palmitic acid; C18:0, stearic acid; C18:1n-9, oleic acid; C18:2n-6, linoleic acid; C18:3n-3, α-linolenic acid; C20:4n-6, arachidonic acid; C20:5n-3, eicosapentaenoic acid (EPA); C22:6n-3, docosahexaenoic acid (DHA). Columns having the same letter in same type are not significantly different (*p* ≥ 0.05).

RBC lipid also reflects the long period dietary fat intake (Dougherty et al., 1987). Fig. 3a, b, and c illustrate the main fatty acid profile change of mice RBC in group C, group N, and group G, respectively. As group C continuously received a soybean oil supplemented diet, their RBC fatty acid profile remained stable throughout the 13-week intervention. In terms of flaxseed oil, proportions of oleic acid (C18:1n-9) and linoleic acid (18:2n-6) progressively declined over time. Correspondingly, the proportion of ALA increased, consistent with the findings in adipose tissue. At the end of dietary intervention, fatty acid content of 3 groups was summarized in Fig. 3d. At week 13, mice fed UGFO or GFO exhibited significantly higher proportions of ALA and EPA and lower proportions of oleic acid and linoleic acid in RBC lipids than those in group C (Fig. 3d). However, neither DHA nor arachidonic acid (C20:4n-6, ARA) proportions differed significantly among the three groups. Thus, the increase in RBC n-3 fatty acids was accompanied by a reduction in linoleic acid, but not ARA, indicating that individual n-6 fatty acids responded differently to the dietary interventions. Our findings were in accordance with those of previous studies. Fan et al. (2020) found that mice fed ALA enriched butter showed higher levels of ALA and EPA in RBC. In terms of human dietary intervention, Edel et al. (2016) observed increased plasma ALA and EPA concentrations following 30 days of flaxseed consumption. Similarly, in the study of Harper et al. (2006), plasma ALA, DPA, and EPA concentrations significantly increased after 12 weeks supplementation of flaxseed oil capsules, whereas olive oil capsules produced no such effects. All aforementioned studies had not observed DHA level increase either in RBC or plasma. Moreover, similar to result of adipose tissue, content of ALA and EPA did not show significant difference between group N and group G. The lower ALA proportion in GFO was not accompanied by a detectable reduction in RBC ALA or EPA proportions under the present experimental conditions. Brain lipidomics was subsequently performed to examine whether the dietary interventions were associated with changes in EPA- and DHA-containing lipid species.

### 3.6 Lipomics analysis to brain lipids

Brain lipidomics was performed to characterize lipid compositional responses to the dietary oils. A total of 30 lipids species were identified with phosphatidylcholines (PC) being the most abundant class. The lipid class distribution is presented in Fig. 4a. The detected lipids distribution was consistent with previous study (Yu et al., 2020). Result of principal component analysis (PCA) to these lipids is depicted in Fig. 4b. Dimensionality reduction to 2 principal components showed overlapping distributions for groups N and G and separation from group C, suggesting a shared pattern of lipidomic variation in the flaxseed oil groups. EPA- and DHA-containing lipid species were then examined individually (Fig. 5). Eighteen EPA and DHA containing lipid species were identified, comprising: 1 phosphatidylglycerol (PG, C22:6), 1 free fatty acid (FA, C22:6), 3 phosphatidylethanolamines (all PE, C22:6), 3 alkyl PE (PE O, 1 C20:5 and 2 C22:6), 3 phosphatidylinositols (PI, 1 C20:5 and 2 C22:6), 1 phosphatidylserine (PS, C22:6), 4 phosphatidylcholines (PC, 1 C20:5 and 3 C22:6), and 2 diacylglycerols (all DG, C22:6). The type and content of DHA was higher than those of EPA, indicating that DHA was rich in brain compared with RBC. Concentrations of other lipids is attached in supplementary material (Fig. S2).

**Fig. 4:**
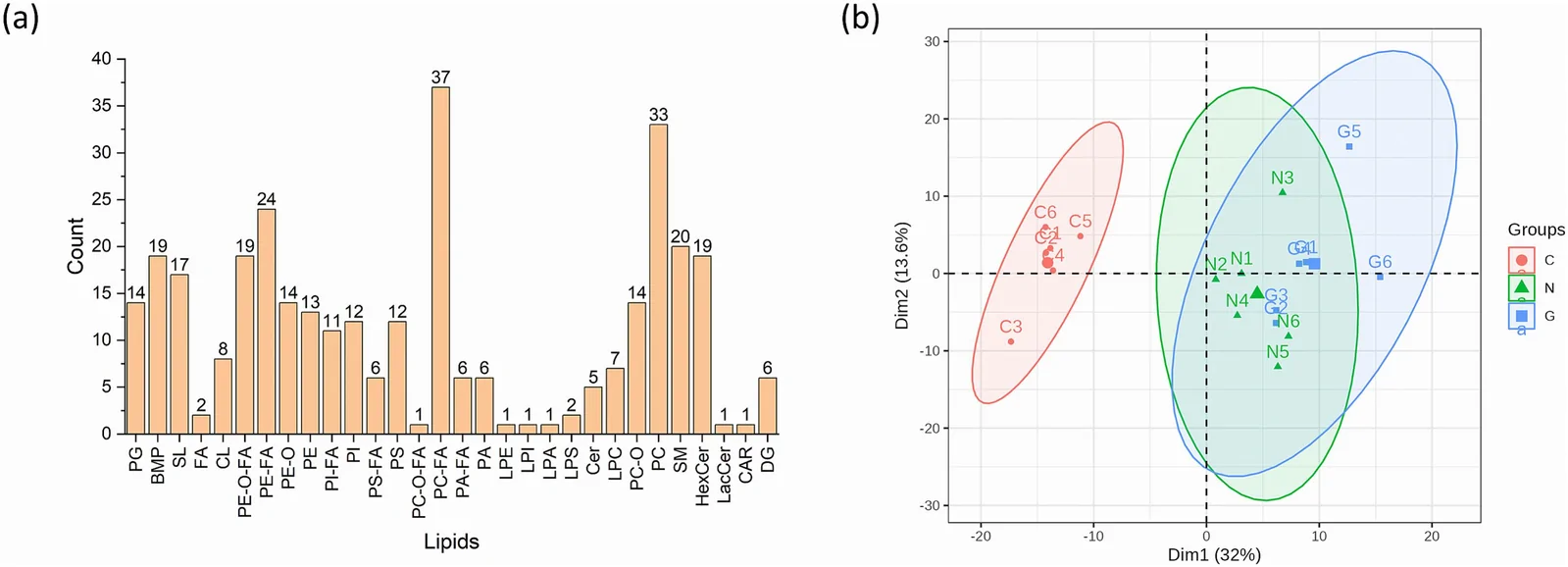
Results of lipid types (a) and PCA score plot (b) of mice brain **lipidomics**. Ellipses in (b) are confidence ellipses of 95% confidence level.

**Fig. 5:**
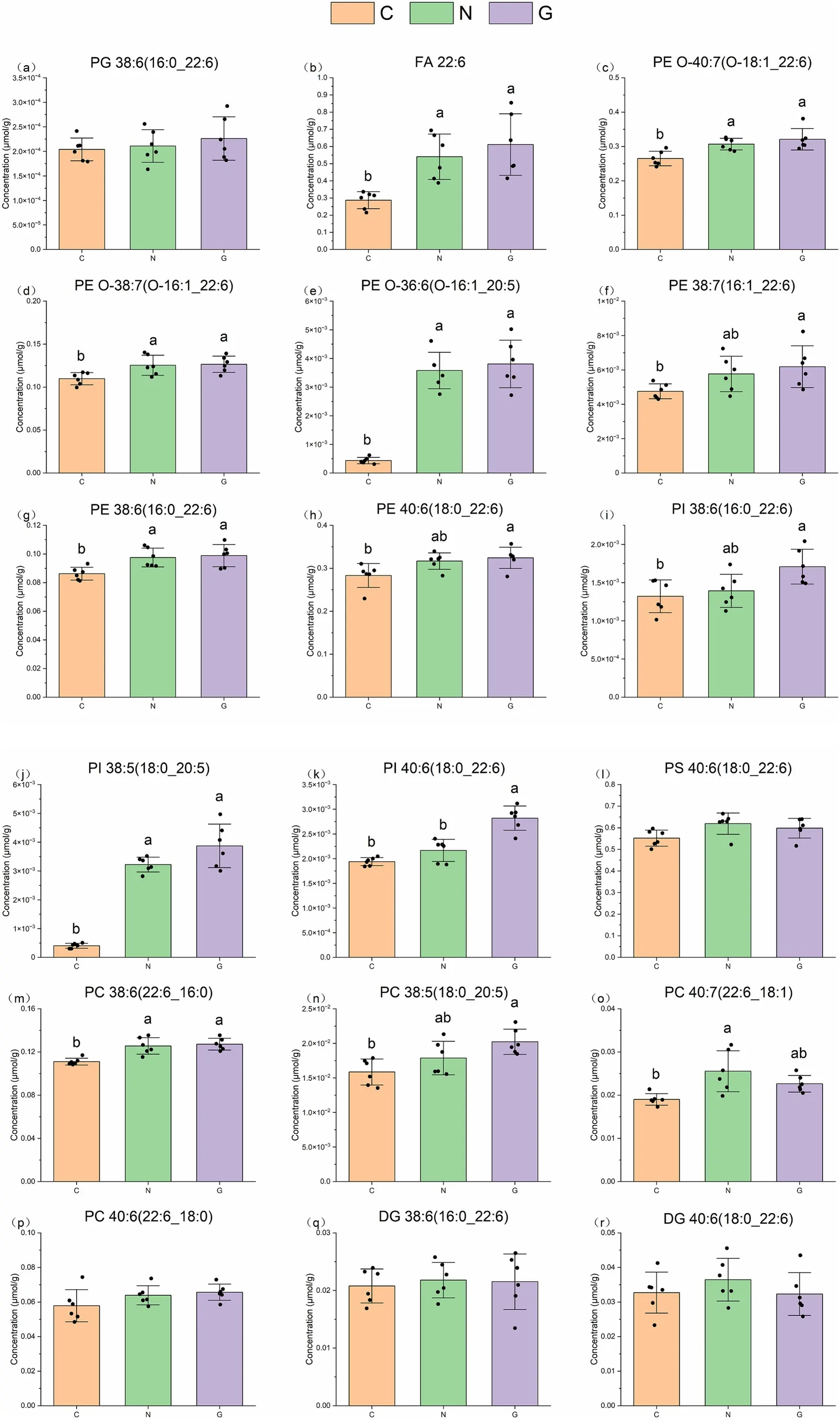
Concentrations of phosphatidylglycerol (PG) 38:6(16:0_22:6) (a), free fatty acid (FA) C22:6 (b), alkyl PE (PE O)-40:7(O-18:1_22:6) (c), PE O-38:7(O-16:1_22:6) (d), PE O-36:6(O-16:1_20:5) (e), phosphatidylethanolamine (PE) 38:7(16:1_22:6) (f), PE 38:6(16:0_22:6) (g), PE 40:6(18:0_22:6) (h), phosphatidylinositol (PI) 38:6(16:0_22:6) (i), PI 38:5(18:0_20:5) (j), PI 40:6(18:0_22:6) (k), phosphatidylserine (PS), 40:6(18:0_22:6) (l), phosphatidylcholine (PC) 38:6(22:6_16:0) (m), PC 38:5(18:0_20:5) (n), PC 40:7(22:6_18:1) (o), PC 40:6(22:6_18:0) (p), diacylglycerol (DG) 38:6(16:0_22:6) (q), and DG 40:6(18:0_22:6) (r) in brain between group C, group N, and group G. Columns having the same letter are not significantly different (*p* ≥ 0.05).

Compared with group C, the flaxseed oil groups showed higher concentrations of several EPA- or DHA-containing lipid species (Fig. 5). Most measured species did not differ significantly between groups N and G, whereas PI 40:6(18:0_22:6) was significantly higher in group G than in both other groups, indicating changes in brain lipid composition. The functional consequences of these changes remain to be further established. DHA containing lipids such as DG were not affected by flaxseed oil, this may suggest that existing of these lipids was stable, which was hard to be influenced by variation of dietary lipids.

The observed increases in several DHA-containing phospholipids compared with group C, including PC and PE species, may have implications for neuronal membrane properties and synaptic function. In experimental hippocampal models, DHA supplementation promoted neurite growth, synaptic protein expression, and synaptic activity, whereas developmental n-3 fatty acid deprivation impaired long-term potentiation (Cao et al., 2009). These findings provide a rationale for investigating the functional relevance of the lipid changes observed in this study. Future studies combining brain lipidomics with assessments of synaptic proteins, electrophysiological function, and learning and memory would help determine whether these compositional changes have functional consequences.

### 3.7 General discussion

As a novel and relatively economical pretreatment method, germination can activate a series of metabolic pathways in flaxseed (Dong et al., 2024; Dong et al., 2025), altered both the fatty acid profile and minor-component composition of flaxseed oil, lowering the ALA proportion while increasing sterol and lignan contents. These changes indicate that germination should be evaluated beyond any single compositional endpoint. Previous work documented lipid mobilization and reduced oil yield during germination (Dong et al., 2023, 2025), highlighting a processing trade-off between oil recovery and compositional enrichment. Optimization should therefore balance ALA retention, individual lignan profiles, sterol enrichment, and oil yield.

The dietary intervention revealed changes in tissue fatty acid profiles without significant differences in body weight, adipose tissue weight, or plasma lipid parameters. Relative to soybean oil, both flaxseed oils increased adipose tissue ALA proportions and RBC ALA and EPA proportions, while reducing linoleic acid proportions. RBC ARA and DHA proportions remained unchanged, showing that individual fatty acids responded differently. Despite the lower ALA proportion in GFO, most measured tissue n-3 fatty acid outcomes did not differ significantly between UGFO and GFO. These observations characterize tissue responses to dietary intervention. However, due to the ALA daily intake was not recorded in this study, quantity effects or greater ALA utilization efficiency can not be established yet. Brain lipidomics complemented the peripheral tissue measurements by revealing differences in several EPA- and DHA-containing lipid species relative to the soybean oil control. Most measured species did not differ significantly between the two flaxseed oil groups, except PI 40:6(18:0_22:6) was higher with GFO. This selective response indicates differences in lipid molecular composition as discussed in Section 3.6.

Several mechanisms could contribute to these tissue responses. Lignans may influence lipid metabolism. For example, enterolactone, a metabolite of SECO, altered lipid export and proteins associated with fatty acid oxidation in HepG2 cells (Drygalski et al., 2017). Redox dependent regulation is another possibility: prolonged high-fat feeding was associated with oxidative stress and reduced hepatic desaturase activities despite increased enzyme expression (Zúñiga-Hernández et al., 2025). However, the model used in that study differs from the present intervention, and whether GFO affected redox status or enzyme activity requires further study.

A physiological plateau provides another alternative explanation. Both flaxseed oil diets may have supplied sufficient ALA for tissue incorporation or downstream conversion to become less responsive to the modest difference in oil ALA proportions. A threshold in intestinal absorption is also conceivable but requires separate assessment. Mechanistically, mouse liver microsomal assays showed that DHA inhibited FADS2 activity non-competitively with respect to ALA and identified the second Δ6-desaturation reaction, from 24:5n-3 to 24:6n-3, as rate-limiting under the conditions examined (Valenzuela et al., 2025). These findings support a nonlinear conversion response, particularly for DHA. However, due to the daily ALA intake was not recorded in this study, these explanations cannot be distinguished. Future studies should monitor food intake and combine graded ALA doses with comparisons at matched ALA intakes. Given the liver's substantial PUFA biosynthetic capacity (Valenzuela et al., 2024), hepatic fatty acid profiling, FADS1/FADS2 and ELOVL2/ELOVL5 expression and activity, redox assessments, and stable isotope tracing would help distinguish conversion from catabolism and tissue retention. Targeted lignan and sterol interventions could clarify their contributions, while synaptic and behavioral assessments could test the functional relevance of the brain lipid changes. Together, these approaches would further connect germination optimization with measurable nutritional outcomes.

## 4 Conclusions

In this study, germination altered flaxseed oil composition by increasing lignan and sterol contents while reducing the ALA proportion. Following a 13-week dietary intervention in mice, most measured tissue n-3 fatty acid outcomes did not differ significantly between UGFO and GFO, and brain lipidomics revealed changes in several EPA- and DHA-containing lipid species relative to the soybean oil control. These findings characterize the tissue lipid responses to the two kinds of flaxseed oils, and provides a basis for optimizing germination through a balance between ALA retention and minor component enrichment. Future studies with monitored food intake, matched ALA intakes, and direct measurements of metabolic conversion are needed to clarify the contributions of minor oil components.

## CRediT authorship contribution statement

**Zeqing Huang:** Writing – original draft, Validation, Resources, Methodology, Conceptualization. **Shuai Xu:** Visualization, Methodology, Investigation, Formal analysis. **Yonglin Li:** Writing – review & editing, Writing – original draft, Methodology, Investigation, Conceptualization. **Keqiang Huang:** Software, Resources, Data curation. **Chongyang Liu:** Visualization, Formal analysis, Data curation. **Yuan Gao:** Visualization, Software, Project administration. **Qi Li:** Supervision, Methodology, Data curation. **Xiuzhu Yu:** Resources, Project administration, Funding acquisition, Conceptualization.

## Ethics declaration

This study was conducted in accordance with the ARRIVE (Animal Research: Reporting of In Vivo Experiments) guidelines.

This study was approved by the Faculty Animal Policy and Welfare Committee of Northwest A&F University.

(Approval No. IACUC 2025–0505)

## Declaration of competing interest

The authors declare that they have no known competing financial interests or personal relationships that could have appeared to influence the work reported in this paper.

## Data Availability

Data will be made available on request.

## Appendix A Supplementary data

Supplementary material of Effects of flaxseed germination on oil composition, tissue n-3 fatty acid profiles, and brain lipidomics in mice.
