# Supplementary material for: Effects of flaxseed germination on oil composition, tissue n-3 fatty acid profiles, and brain lipidomics in mice
Source: Food Chem X. 2026 Oct 18;40:104654. doi: 10.1016/j.fochx.2026.104654 (PMC13717004; doi:10.1016/j.fochx.2026.104654)
Supplement: Supplementary file 1 [file mmc1.docx]

**Effects of flaxseed germination on oil composition, tissue n-3 fatty acid profiles, and brain lipidomics in mice**

**Supplementary material**

**Zeqing Huang, Shuai Xu, Yonglin Li*, Keqiang Huang, Chongyang Liu, Yuan Gao, Qi Li, Xiuzhu Yu***

**Table captions**

**Table. S1** Composition of animal chows.

**Table. S2** Fatty acid profiles of the oils incorporated into the animal chows.

**Table. S1** Composition of animal chows.

| Composition | Control | | UGFO chow | | GFO chow | |
| --- | --- | --- | --- | --- | --- | --- |
|  | Weight (g) | Energy (kcal) | Weight (g) | Energy (kcal) | Weight (g) | Energy (kcal) |
| Casein | 200 | 800 | 200 | 800 | 200 | 800 |
| DL-Methionine | 3 | 12 | 3 | 12 | 3 | 12 |
| Corn starch | 18.6 | 74.4 | 18.6 | 74.4 | 18.6 | 74.4 |
| Sucrose | 500 | 2000 | 500 | 2000 | 500 | 2000 |
| Cellulose BW200 | 50 | 0 | 50 | 0 | 50 | 0 |
| Soybean oil | 108.4 | 975.6 | 0 | 0 | 0 | 0 |
| UGFO | 0 | 0 | 108.4 | 975.6 | 0 | 0 |
| GFO | 0 | 0 | 0 | 0 | 108.4 | 975.6 |
| Mineral mixture | 35 | 0 | 35 | 0 | 35 | 0 |
| Vitamin blend | 10 | 40 | 10 | 40 | 10 | 40 |
| Choline bitartrate | 2 | 0 | 2 | 0 | 2 | 0 |
| Red pigment | 0.05 | 0 | 0 | 0 | 0 | 0 |
| Yellow pigment | 0 | 0 | 0.05 | 0 | 0 | 0 |
| Blue pigment | 0 | 0 | 0 | 0 | 0.05 | 0 |
| Total | 927.05 | 3902 | 927.05 | 3902 | 927.05 | 3902 |
|  | Weight (%) | Energy (%) | Weight (%) | Energy (%) | Weight (%) | Energy (%) |
| Protein | 21.9 | 20.8 | 21.9 | 20.8 | 21.9 | 20.8 |
| Carbohydrate | 57 | 54.2 | 57 | 54.2 | 57 | 54.2 |
| Oil | 11.7 | 25 | 11.7 | 25 | 11.7 | 25 |

**Table. S2** Fatty acid profiles of the oils incorporated into the animal chows*^a^*.

| Fatty acid (%) | Soybean oil (Group C) | UGFO (Group N) | GFO (Group G) |
| --- | --- | --- | --- |
| C16:0 | 10.53 ± 0.27 | 5.43 ± 0.08 | 5.55 ± 0.02 |
| C18:0 | 4.41± 0.07 | 3.96 ± 0.04 | 4.85 ± 0.01* |
| C18:1n-9 | 25.66 ± 0.14 | 17.30 ± 0.11 | 20.73 ± 0.03* |
| C18:2n-6 | 51.76 ± 0.62 | 16.14 ± 0.03 | 15.52 ± 0.003* |
| C18:3n-3 | 6.29 ± 0.05 | 57.17 ± 0.27 | 53.35 ± 0.06* |

*^a^*: Values are expressed as percentages of total fatty acids and presented as the mean ± SD. UGFO and GFO data were obtained from three independently prepared oil samples per treatment. Soybean oil data were obtained from oil used to prepare control chow, the oil was sampled for 3 times and determined.

**Figure captions**

**Fig. S1** Experiment design of dietary intervention on mice.

**Fig. S2** heat map of all lipids in mice brain of group C, group N, and group G.

**Fig. S1** Experiment design of dietary intervention on mice.

**Fig. S2** heat map of all lipids in mice brain of group C, group N, and group G.
